# Redox enzyme-mimicking activities of CeO_2_ nanostructures: Intrinsic influence of exposed facets

**DOI:** 10.1038/srep35344

**Published:** 2016-10-17

**Authors:** Yushi Yang, Zhou Mao, Wenjie Huang, Lihua Liu, Junli Li, Jialiang Li, Qingzhi Wu

**Affiliations:** 1 State Key Laboratory of Advanced Technology for Materials Synthesis and Processing, and Biomedical Material and Engineering Center, Wuhan University of Technology, Wuhan 430070, China; 2 School of Chemical Engineering and Life Science, Wuhan University of Technology, Wuhan 430070, China; 3 School of Chemical Engineering, Shangdong University of Technology, Zibo 255000, China

## Abstract

CeO_2_ nanoparticles (NPs) have been well demonstrated as an antioxidant in protecting against oxidative stress-induced cellular damages and a potential therapeutic agent for various diseases thanks to their redox enzyme-mimicking activities. The Ce^3+^/Ce^4+^ ratio and oxygen vacancies on the surface have been considered as the major originations responsible for the redox enzyme-mimicking activities of CeO_2_ NPs. Herein, CeO_2_ nanostructures (nanocubes and nanorods) exposed different facets were synthesized via a facile hydrothermal method. The characterizations by X-ray photoelectron spectroscopy, Raman spectroscopy, and UV-Vis spectroscopy show that the Ce^3+^/Ce^4+^ ratio and oxygen vacancy content on the surfaces of as-synthesized CeO_2_ nanostructures are nearly at the same levels. Meanwhile, the enzymatic activity measurements indicate that the redox enzyme-mimicking activities of as-synthesized CeO_2_ nanostructures are greatly dependent on their exposed facets. CeO_2_ nanocubes with exposed {100} facets exhibit a higher peroxidase but lower superoxide dismutase activity than those of the CeO_2_ nanorods with exposed {110} facets. Our results provide new insights into the redox enzyme-mimicking activities of CeO_2_ nanostructures, as well as the design and synthesis of inorganic nanomaterials-based artificial enzymes.

Enzyme-mimicking activities of various nanomaterials have attracted considerable interest in industrial catalysis and biomedical fields. CeO_2_ nanoparticles (NPs) have been well demonstrated as an antioxidant highly effective in protecting against oxidative stress-induced cellular damages and a potential therapeutic agent for various diseases, such as cancer, diabetes, chronic inflammation, brain autoimmune degenerative disease, and retinal pathologies^1^,^2^,^3^,^4^. Numerous studies have shown that CeO_2_ NPs display high activities mimicking a series of redox enzymes, including superoxide dismutase (SOD)^5^,^6^,^7^, catalase^8^,^9^,^10^, peroxidase^11^,^12^,^13^,^14^,^15^, phosphotriesterase^16^, phosphatase^17^,^18^, and oxidase^19^, which can scavenge reactive oxygen and nitrogen species. In CeO_2_, the intrinsic defects derived from the existence of Ce^3+^ result in the formation of oxygen vacancies compensating for the positive charge deficiency. The enzyme-mimicking activities of CeO_2_ NPs have been attributed to the auto-regenerative cycle of Ce^3+^/Ce^4+^ and oxygen vacancies on the surface of CeO_2_. Hence, the redox state of Ce ions on the surface plays a crucial role in the redox enzyme-mimicking activities of CeO_2_ NPs. Studies by Self *et al.*^5^,^6^,^7^,^8^,^9^,^10^ indicated that the high Ce^3+^/Ce^4+^ ratio on the surface of CeO_2_ NPs corresponded to high SOD mimetic activity, which was inhibited and transferred to catalase/peroxidase mimetic activities when the Ce^3+^/Ce^4+^ ratio decreased. On the other hand, some results suggested that increasing the Ce^3+^ concentration on the surface of CeO_2_ NPs was beneficial to peroxidase mimetic activity^11^,^12^.

The exposed facets have been demonstrated to considerably contribute to the catalytic performances of various metals, alloys, and metal oxides^20^,^21^,^22^,^23^. Recent studies by Shen *et al.* based on theoretical calculations and experimental data confirmed that redox enzyme-mimicking activities of noble metals (Au, Ag, Pd, and Pt) were critically dependent on exposed facets^24^. Previous studies also showed that the catalytic activities of CeO_2_ NPs in the oxidation of CO, NO, and propane were substantially related to the exposed facets^25^,^26^,^27^,^28^,^29^,^30^,^31^. So far, the relationship between the exposed facets of CeO_2_ NPs and their redox enzyme-mimicking activities has not yet been explored.

Herein, CeO_2_ nanocubes with exposed {100} facets and nanorods with exposed {110} facets were synthesized through a facile hydrothermal method. Phase structures and morphologies were characterized by X-ray diffraction (XRD), transmission electron microscopy (TEM), and high resolution transmission electron microscopy (HRTEM). The surface information of the cerium element located on the surface of the CeO_2_ nanostructures was analyzed by X-ray photoelectron spectroscopy (XPS), Raman spectroscopy, and UV-Vis spectroscopy. The specific surface areas of the CeO_2_ nanostructures were measured by nitrogen adsorption in accordance with the Brunauer-Emmett-Teller (BET) method. Furthermore, the peroxidase and SOD mimetic activities of the CeO_2_ nanostructures were evaluated.

## Results and Discussion

**Figure 1:**
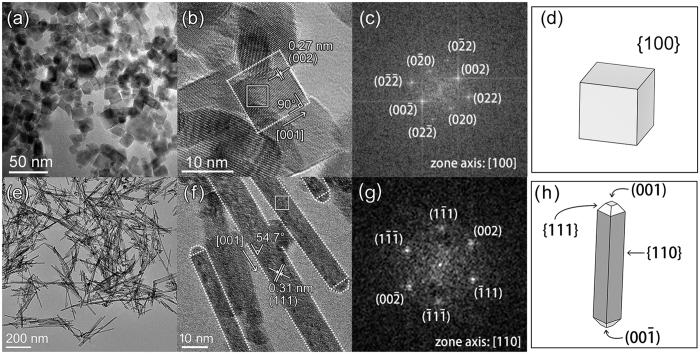
TEM and HRTEM images of the as-synthesized CeO_2_ nanostructures. (**a–f**) CeO_2_ nanocubes; (**e–h**) CeO_2_ nanorods. (**c,g**) show the FFT patterns of the areas marked with white lines in (**b,f)**, respectively. (**e,h**) display the proposed models of the CeO_2_ nanocubes and nanorods, respectively. The CeO_2_ models were drawn using software VESTA^32^.

Figure 1 shows the TEM and HRTEM images of the as-synthesized CeO_2_ nanostructures. Homogeneous CeO_2_ nanocubes were obtained at an average size of approximately 13 ± 3 nm (Fig. 1a). The HRTEM image displays the well-aligned crystal planes, indicating the single-crystalline nature of CeO_2_ nanocubes (Fig. 1b). The interplanar spacing of approximately 0.27 nm could be indexed to the (200) plane of the face-centered cubic CeO_2_. The spots that appeared on the fast Fourier transformation (FFT) pattern of the area marked with white lines in Fig. 1b were indexed, and the zone axis was found to be [100] (Fig. 1c). Accordingly, a three-dimensional model describing the nanocubes is shown in Fig. 1d, representing a cubic structure enclosed with {100} facets. CeO_2_ nanorods with an average diameter of approximately 10 ± 3 nm were obtained by adjusting the synthesis parameters (Fig. 1e). The well-aligned crystal planes observed from the HRTEM image in Fig. 1f also indicate the single-crystalline structure of CeO_2_ nanorods.

The interplanar spacing of approximately 0.31 nm could be indexed to the (111) plane. The spots that appeared on the FFT pattern of the area marked with white lines in Fig. 1f were indexed, and the zone axis was found to be [110] (Fig. 1g). Accordingly, a three-dimensional model describing the nanorods is shown in Fig. 1h, suggesting a rod-like structure enclosed with {110} and {111} facets and truncated by (100) and (00Ī) facets at the z-axis.

**Figure 2:**
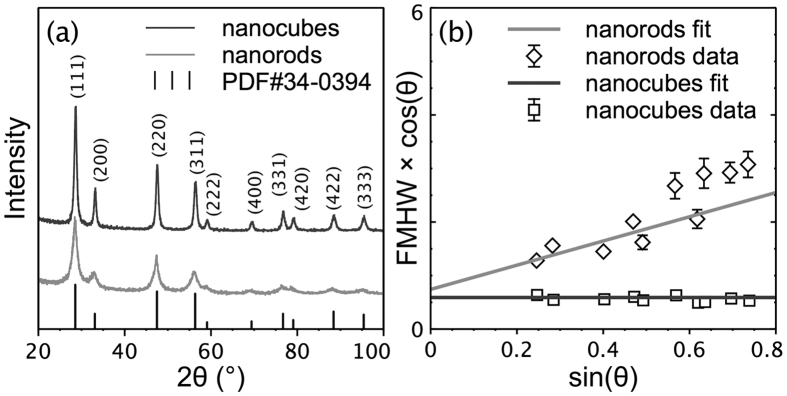
(**a**) XRD patterns of the as-synthesized CeO_2_ nanostructures. (**b**) Williamson-Hall plots of the CeO_2_ nanostructures derived from the XRD patterns. The coefficient of the fitted lines indicates the microstrain (a higher coefficient denotes a higher microstrain), and the intercept of the fitted lines indicates the crystallite size (a higher intercept means a smaller size). The linear and nonlinear fittings were performed using software Fityk^33^.

The phase structure of the as-synthesized CeO_2_ nanostructures was identified by XRD characterization. All of the diffraction peaks in the XRD patterns could be indexed to the cubic fluorite structure of CeO_2_ (JCPDS Card 34–0394) (Fig. 2a). Pawley method^34^ was adopted to obtain the full width at half maximum (FWHM) of the individual peak in the XRD patterns. The result is illustrated as Williamson-Hall plots (Fig. 2b)^35^. The coefficient of the fitted lines reflects the magnitude of the microstrain (a higher coefficient denotes a higher microstrain), and the intercept of the fitted lines indicates the crystallite size. No microstrain existed in the CeO_2_ nanocubes, but a large microstrain was observed in the CeO_2_ nanorods (0% vs. 0.98%). Meanwhile, the crystallite size calculated from the intercept of the fitted lines was approximately 13.5 nm for the CeO_2_ nanocubes and 10.7 nm for the CeO_2_ nanorods, consistent with the average size obtained from the TEM images. In addition, the BET specific surface area measured by N_2_ adsorption-desorption isotherms was approximately 70.9 m^2^/g for the CeO_2_ nanocubes and 95.4 m^2^/g for the CeO_2_ nanorods, indicating the larger specific surface area of the CeO_2_ nanorods compared with that of the CeO_2_ nanocubes.

**Figure 3:**
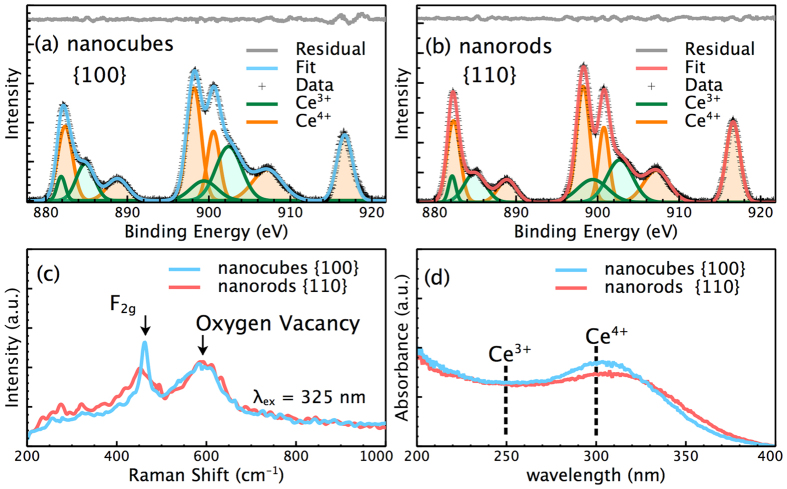
(**a,b**) XPS spectra of the as-synthesized CeO_2_ nanostructures. The peaks in XPS spectra were fitted using Gaussian function and ascribed to Ce^3+^ and Ce^4+^, respectively. (**c**) Raman spectra of the as-synthesized CeO_2_ nanostructures at the excitation wavelength of 325 nm. (**d**) UV-Vis spectra of the as-synthesized CeO_2_ nanostructures.

The surface chemical information of the CeO_2_ nanostructures was analyzed by XPS, Raman spectroscopy, and UV-Vis spectroscopy. The peaks in the XPS spectra were fitted using the Gaussian function (Fig. 3a,b). The areas of the fitting peaks ascribed to Ce^3+^ and Ce^4+^ were used to calculate the contents of Ce^3+^ and Ce^4+^ on the surface of the CeO_2_ nanostructures^15^,^36^. The calculations yielded approximately 31.8% of Ce^3+^ for the CeO_2_ nanocubes and 31.2% of Ce^3+^ for the CeO_2_ nanorods, suggesting the similar Ce^3+^ contents on the surfaces of the as-synthesized CeO_2_ nanostructures. Figure 3c shows the Raman spectra of CeO_2_ nanostructures at the excitation wavelength of 325 nm. The strong peak at 462 cm^−1^ could be ascribed to the F_2g_ mode^37^,^38^. The asymmetrical and broader peak in the Raman spectrum of the CeO_2_ nanorods could be attributed to the smaller crystallite size and larger microstrain compared with those of the CeO_2_ nanocubes^39^. Notably, the two CeO_2_ nanostructures exhibited similar Raman absorption at 595 cm^−1^, which corresponds to the characteristic peak of oxygen vacancy^37^,^38^.

This result implies that oxygen vacancies on the surfaces of both CeO_2_ nanostructures were at the same level. The absorption peak at 250 nm in UV-Vis spectra of CeO_2_ has been ascribed to Ce^3+^, and the absorption peak at 295 nm has been ascribed to Ce^4+ ^6,10. No obvious absorption peak belonging to Ce^3+^ was observed for both of the CeO_2_ nanostructures (Fig. 3d), implying the low Ce^3+^ content in the crystal structure of the CeO_2_ nanostructures. Therefore, these results strongly demonstrated that both the Ce^3+^/Ce^4+^ ratio and oxygen vacancies on the surface of the as-synthesized CeO_2_ nanostructures were nearly at the same levels.

The peroxidase-mimicking activity of the CeO_2_ nanostructures was evaluated by monitoring the catalytic reaction between H_2_O_2_ and tetramethylbenzidine (TMB) in acetic buffer solution (equation 1).


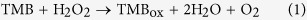


**Figure 4:**
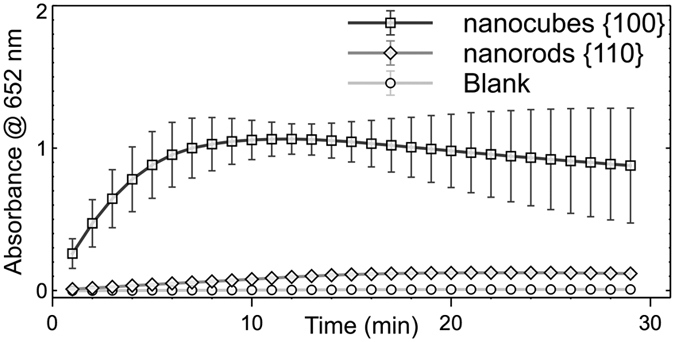
Peroxidase mimetic activity of the CeO_2_ nanostructures. The change in absorbance at 652 nm represents the conversion of TMB to oxidized TMB (TMB_ox_).

The change in absorbance at 652 nm indicates the formation of TMB_ox_. Almost no change in absorbance was observed within 30 min (Fig. 4), implying that H_2_O_2_ failed to oxidize TMB in the absence of catalysts. Subsequently, in the presence of CeO_2_ nanorods with exposed {110} facets, a slight increase in absorbance was observed with reaction time extension, suggesting that the TMB was slowly oxidized by H_2_O_2_. The absorbance was dramatically increased with the reaction prolongation in the presence of CeO_2_ nanocubes, demonstrating that the oxidation of TMB by H_2_O_2_ was greatly accelerated by the CeO_2_ nanocubes with exposed {100} facets.

**Figure 5:**
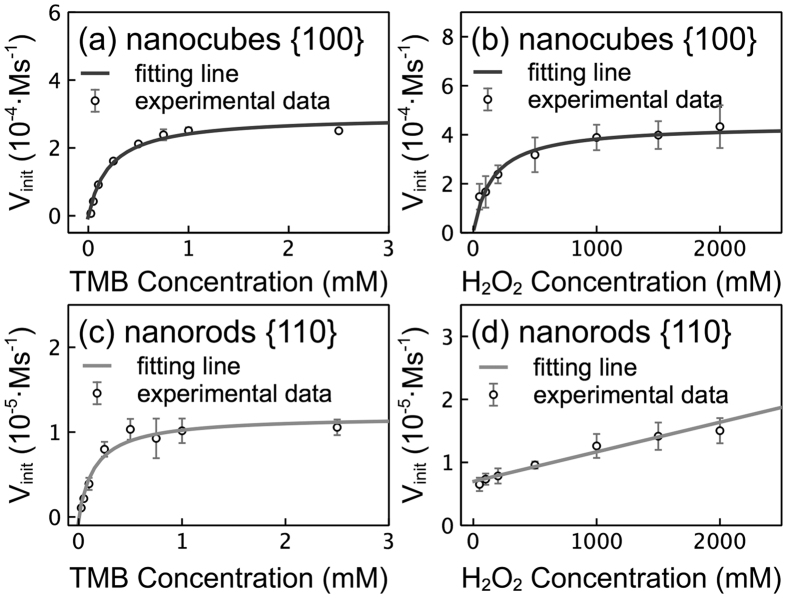
Steady-state kinetic assay of peroxidase mimetic activity.

Figure 5 shows the results of a steady-state kinetic assay conducted by varying the concentrations of TMB and H_2_O_2_. Kinetic parameters were calculated by fitting experimental data to the Michaelis-Menten equation. *V*_max_ is the maximal reaction velocity, and *K*_*m*_ is the Michaelis constant. A higher *V*_max_ value represents a higher conversion rate from substrate to product, and a higher *K*_*m*_ denotes a smaller catalyst affinity to the substrate. Under varied TMB concentrations (Fig. 5a,c), the *K*_*m*_ value was approximately 0.217 mM for the CeO_2_ nanocubes and approximately 0.240 mM for the CeO_2_ nanorods, consistent with the values reported previously^14^,^40^. These results suggest the similar affinities of TMB to both of the CeO_2_ nanostructures. However, *V*_max_ value was approximately 8.2 × 10^−8^ M/s for the CeO_2_ nanocubes and 0.4 × 10^−8^ M/s for the CeO_2_ nanorods, indicating that the rate of the reaction catalyzed by the CeO_2_ nanocubes with exposed {100} facets was 23 times higher than that catalyzed by the CeO_2_ nanorods with exposed {110} facets. Meanwhile, under varying H_2_O_2_ concentrations, the *K*_*m*_ and *V*_max_ for the CeO_2_ nanocubes were approximately 153.6 mM and 12.2 × 10^−8^ M/s, respectively. In such cases, the *K*_*m*_ obtained is far greater than that obtained from the varied TMB levels, suggesting that the affinity of H_2_O_2_ on the surface of the CeO_2_ nanocubes was far smaller than that of TMB. While the larger *V*_max_ in the case of varying H_2_O_2_ concentrations than that in the case of varying TMB concentrations suggests that the catalytic reaction was more sensitive to the H_2_O_2_ concentration. Notably, the Michaelis-Menten equation failed to describe the relation between the concentration of H_2_O_2_ and the initial velocity of the reaction in the presence of the CeO_2_ nanorods (Fig. 5d). Instead, a linear relation was observed, implying a simple first-order reaction dependent on the concentration of the H_2_O_2_ catalyzed by the CeO_2_ nanorods.

The peroxidase-mimicking activity of the CeO_2_ nanorods was also measured after a heating treatment at 650 °C for 48 h because the fitting of the XRD patterns reveals the existence of microstrain in the CeO_2_ nanorods. However, no significant increase in enzyme activity was observed in despite of the disappearance of the microstrain in the CeO_2_ nanorods after annealing (see Supplementary Figs S1 and S2). Hence, the peroxidase mimetic activities of the CeO_2_ nanostructures were influenced by the exposed facets instead of the microstrain.

The SOD mimetic activity of the CeO_2_ nanostructures was also evaluated with colorimetric assay kits. The results indicate that the SOD mimetic activity of the CeO_2_ nanorods with exposed {110} facets was four times higher than that of the CeO_2_ nanocubes with exposed {100} facets (see Supplementary Fig. S3). After annealing to remove the microstrain, the CeO_2_ nanorods still exhibited a higher SOD mimetic activity than that of the CeO_2_ nanocubes. Thus, the SOD mimetic activity of the CeO_2_ nanostructures may have also depended on the exposed facets instead of the microstrain.

Theoretical calculations and experimental data have indicated that redox enzymes-mimicking activities of CeO_2_ could be ascribed to the transformation between Ce^4+^ and Ce^3+^ ions and the generation of oxygen vacancies compensating for the positive charge deficiency on the surfaces^41^,^42^,^43^. However, in the present study, both the Ce^3+^/Ce^4+^ ratio and oxygen vacancies were of the same level on the surfaces of the two CeO_2_ nanostructures, which displayed different redox enzyme-mimicking activities. Therefore, the different exposed facets should be responsible for such different catalytic performances. It is generally known that CeO_2_ possesses three low-index facets: {111}, {110}, and {100} facet. Among which, CeO_2_ {110} surface is a type I ionic crystal surface with neutral atomic planes due to a stoichiometric balance between anions and cations, while CeO_2_ {111} surface is a type II ionic crystal surface with charged planes but without net dipole moment perpendicular to the surface. Both CeO_2_ {110} and {111} surfaces have relatively low surface energies and display modest relaxations on the surfaces compared to the bulk. By comparison, CeO_2_ {100} surface has a nearly infinite free energy and therefore require a major reconstruction compared to the bulk because such a surface consists of alternatively charged planes and thus produces a dipole moment perpendicular to the surface. Accordingly, the stability of three facets decreased in an order of {111} > {110} > {100}, which means the higher catalytic activity of {100} facets than that of {110} and {111} facets^41^,^42^,^43^. According to an energy-based model describing the facet-dependent redox enzyme-mimicking activities of noble metal NPs, the dissociative adsorption of O_2_ on the metal surfaces was proposed to be the key step that provided the surfaces with oxidase-like activities^24^. Meanwhile, the protonation of O_2_^·−^ and adsorption and rearrangement of HO_2_^·^ on metal surfaces were mainly responsible for SOD-like activity of these metals^24^. Therefore, it is reasonable to speculate that the facet-dependent adsorption, activation, and rearrangement processes between the reacting species and the exposed facets may play crucial roles on the redox enzyme-mimicking activities of CeO_2_ nanostructures in addition to the transformation between Ce^4+^ and Ce^3+^ions ratio and the generation of oxygen vacancies on the surfaces. Unfortunately, although numerous theoretical and experimental investigations have been performed in order to interpret the relationship between the catalytic activity and the surface compositions and crystal structures, the accurate atomic structures of CeO_2_ nanostructures, particularly for the CeO_2_ with exposed {110} and {100} facets, still remain indistinct^41^,^42^,^43^,^44^. Therefore, more theoretical and experimental investigations are necessary to elucidate the precise mechanisms on the influences of redox enzyme-mimicking activities of CeO_2_ nanostructures by their exposed facets.

## Conclusion

In summary, we have shown that the redox enzyme mimetic activities of the CeO_2_ nanostructures were determined by the exposed facets. At the same levels of Ce^3+^/Ce^4+^ ions and oxygen vacancies on the surfaces, CeO_2_ nanocubes with exposed {100} facets displayed a higher peroxidase but lower SOD mimetic activity than those of the CeO_2_ nanorods with exposed {110} facets. Therefore, integrated factors, including exposed facets, Ce^3+^/Ce^4+^ ratio, and oxygen vacancy surface content, should be carefully considered when CeO_2_ nanostructures are employed as antioxidant and therapeutic agent for various diseases on the basis of their redox enzyme-mimicking activities. Our results provide new insight into the redox enzyme-mimicking activities of CeO_2_ nanostructures, as well as the design and synthesis of other inorganic nanomaterials-based artificial enzymes.

## Methods

### Materials

Sodium hydroxide (NaOH, 96%), hydrogen peroxide (H_2_O_2_, 30%), acetic acid (99.5%) and cerium nitrate hexahydrate (Ce(NO_3_)_3_·6H_2_O, 99%) were of analytical grade and purchased from Sinopharm Chemical Reagent Co. (Shanghai, China). 3,3′,5,5′-tetramethyl-benzidine (TMB, 98%) was obtained from Aladdin (Shanghai, China). Silicon (SRM™ 640e) was obtained from National Institute of Standards and Technology (NIST). All reagents were used as received without further purification.

### Synthesis of CeO_2_ nanostructures

In a typical synthesis, 20 mL of Ce(NO_3_) _3_·6H_2_O solution (0.1 mol/L) was added dropwise into NaOH solution (0.1 mol/L for nanocubes and 12 mol/L for nanorods). The mixed solution was then transferred and sealed in a 50 mL Teflon-lined stainless steel autoclave, with subsequent heating for 24 h. The heating temperature was kept at 140 °C for nanocubes and 100 °C for nanorods. After the reaction, the precipitate was collected by centrifugation (9000 rpm, 5 min) and washed alternately with ethanol and deionized water several times, and then dried at 60 °C in the air.

### Characterization of CeO_2_ nanostructures

The phase structure of the samples was identified by powder X-ray diffraction (XRD) on a D8 Advance diffractometer using Cu Kα radiation (λ = 1.5418 Å). NIST SRM™ 640e was used to obtain the instrumental broadening profile. X-ray photoelectron spectroscopy (XPS) measurements were performed on a spectrometer (Axis Ultra DLD-600W, Kratos Analytical Ltd.) using Al Kα radiation as the excitation source. The morphologies of the samples were observed using high resolution transmission electron microscopy (HRTEM, JEM-2100F STEM/EDS, JEOL Corp, Japan). The Raman spectra of the samples were recorded by a Raman spectrometer with a 325 nm laser excitation (VERTEX 70, Bruker, Germany). The UV-Vis spectra of the samples were recorded with an ultraviolet–visible spectrometer (Shimadzu Corp., UV-2550 PC).

### Enzyme mimetic activity of CeO_2_ nanostructures

The peroxidase mimetic activity of CeO_2_ was evaluated by monitoring the redox reaction between TMB and H_2_O_2_ in the presence of the CeO_2_ nanostructures. Typically, 1 mL acetate buffer solution (50 mM, pH = 4.0) containing 25 μg CeO_2_, 0.5 μmol TMB, and 1 mol H_2_O_2_ was added in a cuvette. The reaction was monitored using an ultraviolet–visible spectroscopy (Shimadzu Corp., UV-2550 PC) at wavelength of 652 nm. The measurement was recorded at an interval of one minute and the temperature was kept at 25 °C. In order to obtain the apparent kinetic parameters, experiments varying concentrations of the substrates were carried out. The measurement was recorded at an interval of 10 seconds in order to obtain a higher accuracy. All the rest reaction conditions were unchanged. More details on the calculation of apparent kinetic parameters can be found in the Supplementary Information. The SOD mimetic activity of CeO_2_ nanostructures was determined with colorimetric assay kits (Nanjing Jiancheng Bioengineering Institute, China).

### Data process

All the non-linear curve fittings (XRD, XPS and Michaelis-Menten Kinetic) were processed using software Fityk (version 1.3.0)^33^. All the experiments were repeated at least triplicated to obtain the standard deviation.

## Additional Information

**How to cite this article**: Yang, Y. *et al.* Redox enzyme-mimicking activities of CeO_2_ nanostructures: Intrinsic influence of exposed facets. *Sci. Rep.* **6**, 35344; doi: 10.1038/srep35344 (2016).

## Supplementary Material

Supplementary Information
