# Supplementary material for: Redox enzyme-mimicking activities of CeO2 nanostructures: Intrinsic influence of exposed facets
Source: Sci Rep. 2016 Oct 17;6:35344. doi: 10.1038/srep35344 (PMC5066218; doi:10.1038/srep35344)
Supplement: Supplementary Information [file srep35344-s1.pdf]

Supporting information

Redox enzyme-mimicking activities of CeO_2 nanostructures: Intrinsic influence of exposed facets

Yushi Yang[1], Zhou Mao[1], Wenjie Huang[1], Lihua Liu[2], Junli Li[2], Jialiang Li[3] & Qingzhi Wu[1]

[1]State Key Laboratory of Advanced Technology for Materials Synthesis and Processing, and Biomedical Material and Engineering Center, Wuhan University of Technology, Wuhan 430070, China

[2]School of Chemical Engineering and Life Science, Wuhan University of Technology, Wuhan 430070, China. Correspondence and requests for materials should be addressed to Q.W. (email: wuqzh@whut.edu.cn.)

[3]School of Chemical Engineering, Shangdong University of Technology, Zibo 255000, China

Correspondence and requests for materials should be addressed to Q.Z.W. (email: wuqzh@whut.edu.cn)

Michaelise-Menten constant calculations

For the peroxidase mimetic reaction, the Michaelis-Menten kinetic equation was selected to describe the relation between the initial velocities (V_{init}) and their relative substrate concentrations. The concentration of the product was calculated using equation:

$$c = \frac{A}{\varepsilon \cdot \ell}$$

Where, c is the concentration of TMB_{ox}, A is the absorbance measured by the spectrometer, ε is the extinction coefficient, and ℓ is the length of the light path. In our experiments, $\ell = 1$ cm and $\varepsilon = 3.9 \times 10^{-4}\ M^{-1}cm^{-1}$ for TMB_{ox} at 652 nm[1]. The initial velocity (V_{init}) was calculated using equation:

$$V_{init} = \frac{\Delta c}{\Delta t}$$

Where, V_{init} is the initial reaction velocity, c is the concentration of TMB_{ox}, and t is the reaction time. The kinetic parameters, K_m and V_{max}, was determined by fitting V_{init} against substrate concentrations according to the Michaelis-Menten equation. The data points were directly fitted with the equation using Levenberg–Marquardt algorithm.

Annealing of CeO_2 nanorods and their peroxidase mimetic activities

Fig. S1a and b show the TEM and HRTEM of CeO_2 nanorods with {110} facets after annealing. It is clear that annealing treatment did not change the morphology and exposed facets of the CeO_2 nanorods. Fig. S1c shows the Williamson-Hall plots of CeO_2 nanorods before and after annealing. The fitted line of CeO_2 nanorods after annealing displayed a zero slope, suggesting that the microstrain existed in the CeO_2 nanorods disappeared after annealing.

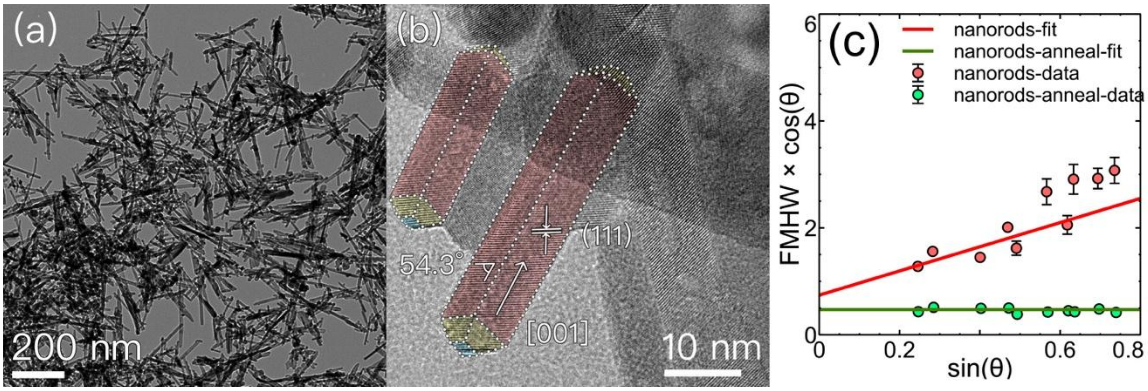

Figure S1. a) TEM image shows uniform CeO_2 nanorods; b) HRTEM image shows the exposed {110} facets. The proposed 3D models were outlined in the image; c) Williamson-Hall plot of CeO_2 nanorods before and after annealing. The slope of the line indicates the microstrain (a larger slope represents a larger microstrain), and the intercept indicates the crystallite size (a large intercept means a smaller size).

Fig. S2 shows the peroxidase mimetic activity of CeO_2 nanorods with {110} facets before and after annealing. It is obvious that annealing did not increase the enzyme activity of CeO_2 nanorods, suggesting that the peroxidase mimetic activity of CeO_2 nanorods was independent on the microstrain.

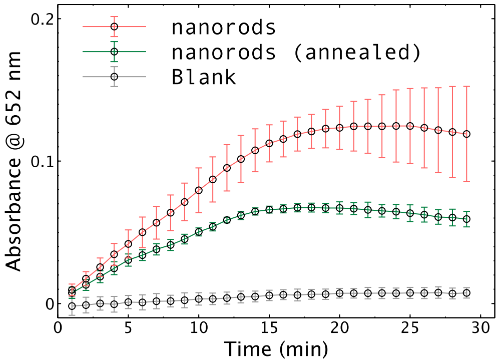

Figure S2. Peroxidase mimetic activity of CeO_2 nanorods before and after annealing. The changes in absorbance at 652 nm represents the conversion from TMB to oxidized TMB (TMB_{ox}).

SOD mimetic activities of the CeO_2 nanostructures

Fig. S3 shows the SOD mimetic activity of CeO_2 nanostructures. Compared with the peroxidase-like activities, CeO_2 nanorods with exposed {110} facets exhibited higher SOD mimetic activity than that of CeO_2 nanocubes with exposed {100} facets. The SOD activity of CeO_2 nanorods was 57.1 U/mg, which was 4 times higher than that of CeO_2 nanocubes. The SOD mimetic activity of CeO_2 nanorods was slightly decreased after annealing, but it was still significantly higher than that of CeO_2 nanocubes. These results indicate that the difference in SOD mimetic activity of CeO_2 nanostructures also originated from the exposed facets, instead of the microstrain.

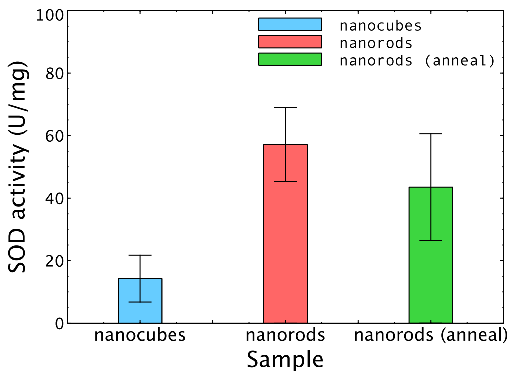

Figure S3. SOD mimetic activity of CeO_2 nanocubes and CeO_2 nanorods (before and after annealing).

Reference

(1) Josephy, P. D., Eling, T. & Mason, R. P. The Horseradish Peroxidase-Catalyzed Oxidation of 3, 5, 3', 5'-Tetramethylbenzidine. *J. Biol. Chem.* **257**, 3669-3675 (1982).
